# Micro-extraction method for the analysis of flame retardants in dust collected from air filters from HVAC systems

**DOI:** 10.1016/j.mex.2024.102693

**Published:** 2024-04-08

**Authors:** Morgan L. Schachterle, Luis E. Lowe, Christopher R. Butler, Allen M. Schoffstall, Janel E. Owens

**Affiliations:** a Department of Chemistry and Biochemistry, University of Colorado Colorado Springs, 1420 Austin Bluffs Parkway, Colorado Springs, CO 80918, United States; b US Geological Survey, Integrated Water Chemistry Assessment Laboratory, 3215 Marine St., #E127, Boulder, CO 80303, United States; c Bayer U.S. – Crop Science, Select Chemistry, Central Lab, 2500 Wiggins Rd., Muscatine, IA 52761, United States

**Keywords:** Flame retardants, Organophosphate esters, Dust, HVAC air filters, Chromatography, Mass spectrometry, Extraction of Flame Retardants from HVAC Air Filter Dust Samples with Analysis by Gas and Liquid Chromatography-Mass Spectrometry

## Abstract

Dust is a sink for many semi-volatile compounds including flame retardants of the organophosphate ester (OPE) and brominated flame-retardant (BFR) classes. Given the large amount of time that we spend indoors, our exposure to these compounds via dust is of significant interest. Here, we present a novel microextraction approach to determine quantitative levels of selected OPEs and BFRs sampled from residential air filters from HVAC systems using a small volume of solvent. Dust samples (25 mg) is extracted with 1 mL of hexane/acetone (50/50, v/v). Upon solvent extraction of these HVAC dust samples, the analytes (TCPP, TDCPP, TPHP, T24DtBPP, TBBPA, and TriBBPA) were quantified via gas chromatography-mass spectrometry (GC/MS) or liquid chromatography-mass spectrometry (LC/MS). The methods for extracting these compounds from HVAC dust samples are detailed here with extensive method validation data to demonstrate accuracy and precision of these methods.

• Dust is a sink for many semi-volatile compounds, including novel or emerging indoor pollutants like the organophosphate ester flame retardant T24DtBPP.
• Here, a small amount of dust (25 mg) is extracted with a small volume of solvent (1 mL hexane and acetone) prior to analysis via chromatographic separation and mass spectrometric detection.

## Graphical abstract

**Figure:**
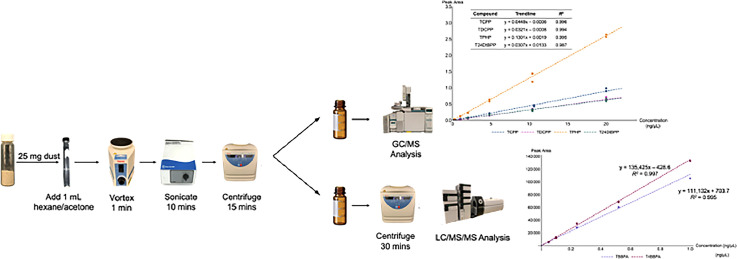

Specifications table

**Table:** 

Subject area	Chemistry
More specific subject area:	Analytical chemistry
Name of your method:	Extraction of Flame Retardants from HVAC Air Filter Dust Samples with Analysis by Gas and Liquid Chromatography-Mass Spectrometry
Name and reference of original method:	Not applicable
Resource availability:	Gas chromatography-mass spectrometry instrument
	Liquid chromatography-mass spectrometry instrument

**Method details**

## Introduction

There is a research gap in analyzing certain OPE and BFR levels in dust samples collected from residential spaces within the U.S. The focus of the work presented here is to demonstrate the applicability of a microextraction approach where 25 mg of dust taken from HVAC filters from residential spaces is extracted with 1.0 mL of solvent with analysis by chromatographic separation and mass spectrometric (MS) detection, including gas chromatography – MS (GC/MS) and liquid chromatography – MS (LC/MS).

## Standards, solvents, and materials

Analytical standards for tris(2-chloro-1-methylethyl) phosphate (TCPP; 66% purity), tris(1,3-dichloro-2-propyl) phosphate (TDCPP; 95% purity), triphenyl phosphate (TPHP; 96% purity), 3,3’,5,5’-tetrabromobisphenol-A (TBBPA; 97% purity), hexamethylbenzene (HMB; 99% purity), and LC/MS-grade ammonium formate were from Sigma-Aldrich (St. Louis, MO). Tris(2,4-di-tert-butylphenyl) phosphate (T24DtBPP; 99.8% purity) was from Toronto Research Chemicals (Toronto, Ontario, Canada). Tribromobisphenol-A (TriBBPA) was synthesized at UCCS.

Certified ACS-grade acetone and hexanes (95% purity) were from Fisher Scientific (Waltham, MA) and Acros Organics (Pittsburgh, PA), respectively. Optima LC/MS-grade methanol was from Fisher Scientific. LC/MS-grade ammonium hydroxide was from Honeywell, Inc. (Morristown, NJ). HPLC-grade 18 MΩ DDI water was produced via a Barnstead e-Pure system (Thermo Fisher Scientific). All other chemicals were from Fisher Scientific unless otherwise stated.

## Synthesis and characterization of TriBBPA

TriBBPA, or 2,6-dibromo-4-[1-(3-bromo-4-hydroxyphenol)-1-methylethyl]phenol, was synthesized by mixing bisphenol-A (4 mmol, 928 mg) with 30 mL methanol and molecular bromine (12 mmol, 620 µL) in a 50 mL round bottom flask at 25°C for 1 h until the orange coloration disappeared. The reaction mixture pH was neutralized by adding 1 M NaOH dropwise (final pH ≧ 6). Solvent was removed from the aqueous methanol solution in vacuo. To the remaining aqueous layer, 30 mL water were added prior to extraction using ethyl acetate (HPLC-grade; 3 extractions with 20 mL aliquots). The combined organic layers were washed with brine, dried with anhydrous Na2SO4, and filtered. The filtrate was evaporated to dryness in vacuo. The resulting crude white solid was purified by column chromatography using a 40 Å fine mesh silica dioxide adsorbent. Column fractions containing the product and byproducts were combined according to TLC analyses and the combined fractions were evaporated in vacuo. The product and two byproducts were observed via TLC and further purified via rotary chromatography. The product (TriBBPA) had an observed Rf value of 0.51 in a 7/3 methylene chloride/hexanes (v/v) chamber. The synthesis yielded 184 mg of TriBBPA (9.9% yield), 1.0 g of TBBPA (∼50% yield) and a small amount of a second byproduct (Rf = 0.38).

The 1H NMR spectrum for the purified TriBBPA was recorded using a Varian Inova 400 MHz NMR spectrometer with resulting chemical shifts (δ, ppm) relative to TMS: 10.1 (OH, s), 9.8 (OH, s), 7.3 (H2’, H2, H6, s), 7.0 (H5’, dd), 6.9 (H6’, d), 1.6 (CH3, s). Following characterization by NMR, the purified TriBBPA was diluted to 100 mg/L in methylene chloride/acetic anhydride (80/20, v/v) and derivatized upon heating at 75°C for 15 min. A 1 µL volume of the derivatized sample was analyzed using a Hewlett Packard 6890 gas chromatograph coupled to a 5973 mass selective detector (GC/MS) used in the full scan (m/z 200 – 600) mode. By derivatizing TriBBPA, the molar mass increased from 464 g/mol (native TriBBPA) to 548 g/mol (TriBBPA + 2 acetyl groups). The resulting GC/MS spectrum [relative intensity, %, and proposed fragment identity] contained: 548 [0.1, M^+^˙]; 506 [21%, (M-acetyl)^+^]; 464 [83%, (M – 2 × acetyl)^+^˙]; 449 [100%, (M – 2 × acetyl – CH3)^+^]; 427 [6%, (M – acetyl – Br)^+^˙]. Purity was determined to be 99.8% with a 0.2% relative impurity from the TBBPA. Finally, the synthesized TriBBPA was analyzed via LC/MS using selected ion monitoring mode where ions for TBBPA (m/z 540.80, 542.80, 544.80) and TriBBPA (m/z 460.90, 462.90, 464.90, 466.90) were monitored. TBBPA eluted at ∼1.2 min and TriBBPA eluted at ∼3.1 min using the column conditions described in Instrumentation and analysis of BFRs – LC/MS. The retention time and isotopic pattern ratio for the TBBPA impurity matched the analytical standard. Experimental isotopic pattern ratios for the TriBBPA pseudomolecular ions matched the expected 3 Br pattern very well (m/z, % abundance): 460.90 (34%), 462.90 (100%), 464.90 (97%), 466.90 (32%).

## Preparation of stock solutions and analytical standards – OPEs

Stock solutions of the organophosphate ester (OPE) flame retardants including 510 ng/µL (TCPP) and 860 ng/µL TDCPP, 1020 ng/µL TPHP, 978 ng/µL T24DtBPP and 980 ng/µL HMB, the internal standard (IS), were prepared in 25.00 mL of hexane/acetone (50/50, v/v) via volumetric flask. (Note: these final concentrations reflect the % purity of the original chemical purchased in Standards, solvents, and materials). Stock solutions were prepared every six months. These individual stock solutions were used to prepare 25.00 mL of a working stock solution containing 10 ng/µL TCPP, TDCPP, TPHP, and T24DtBPP in hexane/acetone (50/50, v/v) via volumetric flask. Separate HMB working stock solutions with concentrations of 10 ng/µL and 0.13 ng/µL were prepared in hexane/acetone (50/50, v/v). The working stock solution of the OPE mix at 10 ng/µL and the HMB IS were used to prepare analytical standards to generate calibration curves (Table 1), and these working stock solutions were prepared every two months. All calibration standards contained a final volume of 0.050 ng/µL HMB. Stocks, working stocks, and analytical calibration standards were stored in glass vials at 3.5°C when not in use.

**Table 1:** Volumes of solvent and working stock solutions to prepare analytical calibration standards for analysis of selected OPEs by gas chromatography-mass spectrometry (GC/MS). All analytical standards contained a final volume of 0.0500 ng/µL HMB, which was added as internal standard (IS).

Concentration of OPEs (ng/µL)	Volume of Hexane/Acetone (50/50, v/v), µL	Volume of OPEs Working Stock (10 ng/µL), µL	Volume of HMB Working Stock (10.0 ng/µL), µL
1.0	900.	100.	5.0
0.52	900.	50.	5.0
0.24	1000.	25.	5.0
0.10	1000.	10.	5.0
0.050	1000.	5.0	5.0
0.010	1000.	1.0	5.0

## Preparation of stock solutions and analytical standards – BFRs

A stock solution of 1350 mg/L of TBBPA in methanol was prepared in a 25.00 mL volumetric flask. TriBBPA was synthesized as described (see Synthesis and characterization of TriBBPA), and a stock solution of 1190 ng/µL TriBBPA in methanol (25.00 mL) was prepared from this synthesized and characterized product. Two working stock solutions containing TBBPA and TriBBPA in methanol (25.00 mL) at 1.0 ng/µL and 10 ng/µL were prepared from the stock solutions, and these working stock solutions were prepared every two months. The 10 ng/µL working stock solution was used for preparing the analytical standards (Table 2). An aliquot of ammonium hydroxide was added to all analytical standards to assist in ionization of the compounds via electrospray ionization (ESI). The stocks, working stocks, and analytical standards of the two BFRs were stored in glass vials at 3.5°C when not in use.

**Table 2:** Volumes of solvent and working stock solutions to prepare analytical calibration standards for analysis of TBBPA and its derivative, TriBBPA, by liquid chromatography-mass spectrometry (LC/MS).

Concentration of BFRs (ng/µL)	Volume of Methanol µL	Volume of BFRs Working Stock (10 ng/µL), µL	Volume of NH_4_OH, µL
1.0	900.	100.	5.0
0.52	900.	50.	5.0
0.24	1000.	25.	5.0
0.10	1000.	10.	5.0
0.050	1000.	5.0	5.0

## Collection and removal of dust from HVAC air filters

HVAC air filters were collected from homes in Colorado Springs, CO (an urban environment) and Berthoud, CO (a rural environment). Because no data regarding demographics or personal information was collected, the study was exempt from IRB review upon discussion with the University's Office of Sponsored Programs and Research Integrity. Participants were, however, asked to fill out a brief online survey to answer questions about age of their home, type of home (standalone vs townhome or apartment), majority of flooring type in the home, frequency of vacuuming, number of electronics in the house, and presence of pets in the home.

Dust was removed from the HVAC air filters by first removing the cardboard frame. The polymeric or fabric membrane of the filter was isolated from any supports added by the manufacturer to maintain the structural integrity of the filter, whether it was metal mesh, plastic webbing, or cardboard. The isolated filter membrane was trimmed to 12 cm × 12 cm squares. The dust was removed from the filter membrane by rubbing the membrane across the surface of a standard test metal sieve with 250 µm openings. The dust was captured beneath the sieve onto a creased clean piece of standard copy paper. On occasion, large debris like pet hair were inadvertently transferred to the sieved dust: this debris was removed with forceps as necessary. The dust was transferred to a tared 40 mL glass vial. The final dust amount (g) was recorded, and vials were stored at room temperature (20°C) until analysis. The sieve and cutting tools were rinsed with methanol between samples to minimize contamination between samples.

## Sample preparation

Approximately 25 mg of sieved dust was weighed (Mettler Toledo XS64 analytical balance) into a silanized glass culture tube with exact weight recorded. To each tube, 1.0 mL hexane/acetone (50/50, v/v) was added. Tubes were capped with a Teflon-lined cap and vortexed for 1 min. Samples were sonicated for 10 min in an ultrasonic water bath at 25 – 30°C. Samples were centrifuged (Fisher Scientific FS20) for 15 min or until the dust was clearly pelleted from the solvent layer. Upon centrifugation, 10. µL of the dust extract, 20. µL of the 0.13 ng/µL HMB IS solution, and 20. µL hexane/acetone (50/50, v/v) were collectively added to a 350 µL pulled-point glass insert in a crimp-top glass autosampler vial. Vials were vortex-mixed for ten seconds. These vials were transferred to the autosampler tray of the GC/MS and analyzed (see Instrumentation and analysis of OPEs – GC/MS for details).

When the analyte class included the BFRs for analysis by LC/MS, 200 µL of the dust extract were added to a 300 µL PolySpring® insert. These glass inserts were placed in a plastic microcentrifuge vial and centrifuged at 14,700 rpm for 30 min (Thermo Scientific Legend 17 centrifuge). The supernatant was transferred to a clean insert, leaving any particulates behind that may have clogged the PEEK lines of the LC/MS. The insert containing the centrifuged extract was placed in a screw-top autosampler vial and analyzed via LC/MS (see Instrumentation and analysis of BFRs – LC/MS for details).

All samples were analyzed by GC/MS or LC/MS on the same day that they were extracted from the air filter dust. The remaining solvent extract was stored in crimp top glass vials at 3.5°C in case samples needed to be re-analyzed on an instrument. The silanized glass culture tubes that were used for the initial solvent extraction step were triple rinsed with acetone and DI water and baked in a GC oven (250°C for 30 min) so that they could be reused for subsequent extractions.

## Instrumentation and analysis of OPEs – GC/MS

The four OPEs of this study were analyzed via GC/MS given that all were semi-volatile, required no derivatization prior to analysis, and were less than 800 Da. Many of these compounds are common plasticizers as well, and our LC/MS/MS had large interferences for T24DtBPP and TCPP upon analyzing methanol blank and air blank samples. Many research groups utilized LC/MS for the analysis of these compounds, and we urge caution when developing methods on a system that is fitted with many plastic solvent transfer lines or components. In our laboratory, analysis of selected OPEs was not feasible by LC/MS/MS given the high levels of these plasticizers inherent to our system.

A Hewlett-Packard 6890 GC coupled with a 5973 mass selective detector (MSD) was utilized for all analyses. The GC was equipped with HP-5MS capillary column (25 m × 0.200 mm, 0.33 µm film thickness). Hydrogen gas was used as the carrier gas with a column head pressure of 5.05 psi (flow rate of 0.7 mL/min). The inlet was operated in split/splitless mode at 250°C. After injecting 1.0 µL of standard, blank, or sample, the OPEs were separated using a temperature gradient. The initial column temperature was 60°C (hold for 3.00 min) and was ramped to 305°C at 30.00°C/min before holding for 13.00 min. The total run time was 24.17 min.

The MSD was operated in selected ion monitoring (SIM) mode with four acquisition windows with the following temperature zones: 280°C for transfer line, 230°C for the source and 150°C for the quadrupole. The electron multiplier was set at 200 V higher than the tune voltage. The first acquisition window included ions of interest (see Table 3) for TCPP and HMB, window 2 for TDCPP, window 3 for TPHP, and window 4 for T24DtBPP. For each OPE, one quantitation ion and three confirmation ions were monitored. For the HMB, which served as the IS, one quantitation and one confirmation ion were monitored.

**Table 3:** GC and MSD conditions for the analysis of OPEs and IS.

Analyte	Retention Time (min)	SIM Acquisition Window (min)	Quantitation Ion, *m/z*	Confirmation Ion(s), *m/z*	Ion Dwell Time (ms)
HMB (IS)	7.8	6.00 – 9.50	147.1	117.0	40
TCPP	9.1	6.00 – 9.50	277.0	201.0 279.0	40
TDCPP	10.9	9.50 – 11.00	381.0	191.0 209.0 383.0	50
TPHP	11.1	11.00 – 12.00	326.0	215.0 233.0 325.0	50
T24DtBPP	19.1	12.00 – 24.17	647.5	648.4 662.5 663.4	80

**Fig. 1:**
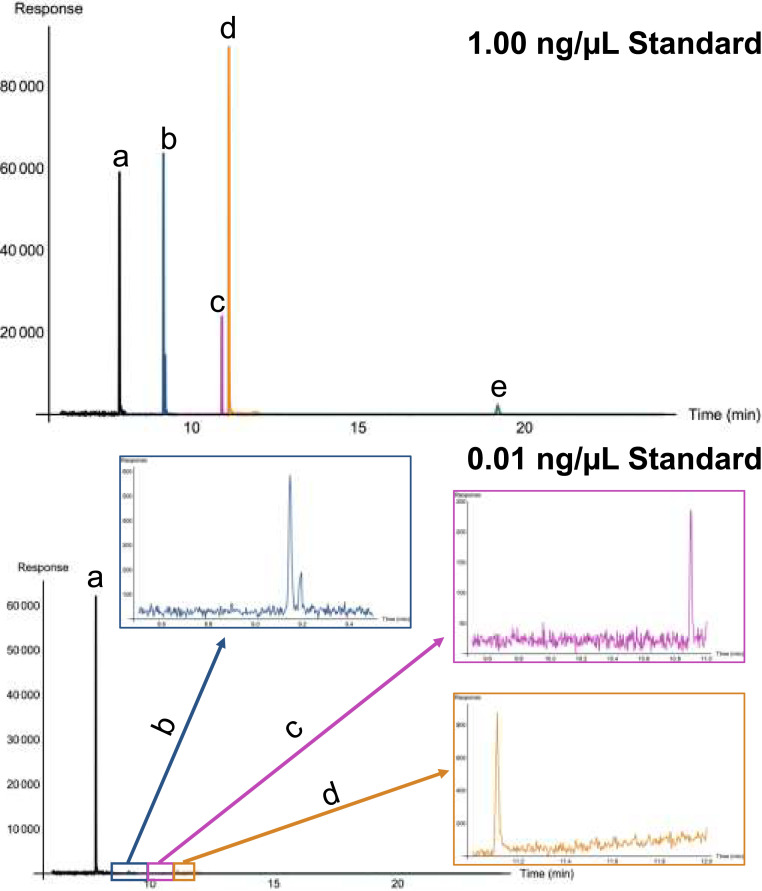
Extracted Ion Chromatograms (EICs) of OPEs included in the study: **a**: HMB (IS), **b**: TCPP, **c**: TDCPP, **d:** TPHP, and **e:** T24DtBPP at two calibration levels: 1.0 ng/µL and 0.010 ng/µL. For the lower calibration standard, the baseline and peak for each analyte excluding T24DtBPP, which was not detected at this level, are shown.

Extracted ion chromatograms (EICs) for 1.00 ng/µL and 0.010 ng/µL analytical standards are included in Fig. 1. The 0.010 ng/µL standard includes enlarged peaks for TCPP, TDCPP, and TPHP owing to the intensity of the HMB peak. The T24DtBPP was not detected at the 0.010 ng/µL level.

### Slope sensitivity and precision across the calibration range

**Fig. 2:**
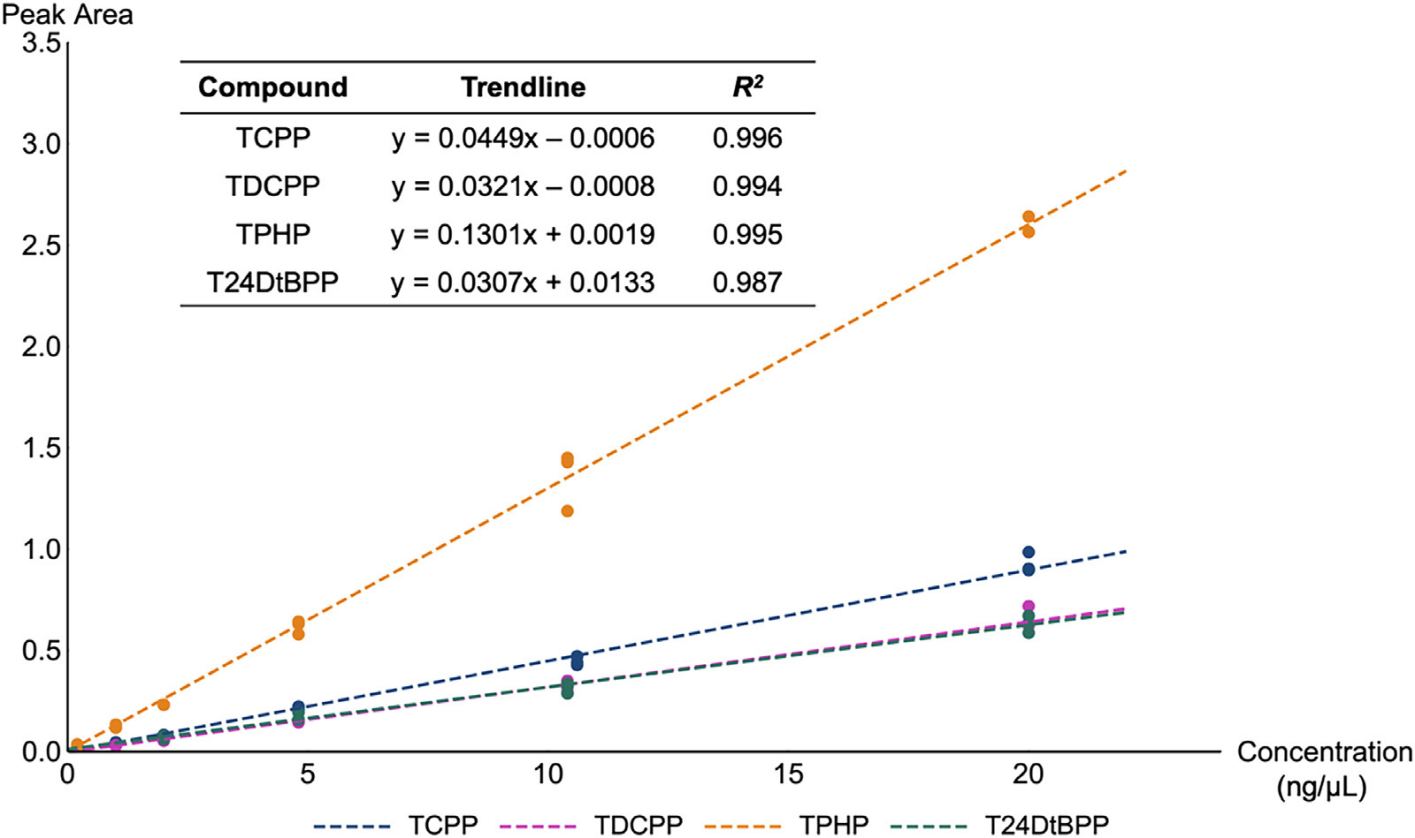
Example of 1/*x* weighted, internal calibration curves used for quantitation of four OPEs via GC/MS. The dark pink TDCPP data and the dark green T24DtBPP data were overlapping given their similar trendline equations.

Calibration standards for OPEs ranged from 0.010 ng/µL to 1.0 ng/µL (Table 1). All quantitation was completed using an internal calibration curve with 1/*x* weighting (Fig. 2). Calibration curves were considered acceptable if the *R^2^* value was greater than 0.97 for averaged curves.

All calibration curves used for quantitation of OPEs extracted from HVAC air filter dust (*n* = 13 generated curves) were recorded with 95% confidence intervals (CIs) calculated to assess the precision of the measurement associated with analytical standards across multiple days (Table 4). The intervals were fairly narrow for all OPEs, suggesting that the calibration curves generated for multiple batches were precise.

**Table 4:** Calibration curves (*n* = 13) using an internal standard calibration model for OPEs with 95% CI included for the slope (*m*) and intercept (*b*). Calibration curves were fit with a linear regression line with 1/*x* weighting.

Analyte	Regression Equation	Mean *R^2^*
TCPP	*y* = (0.04064 ± 0.00290)*x* – (0.00070 ± 0.00061)	0.99
TDCPP	*y* = (0.02076 ± 0.00485)*x* + (0.00006 ± 0.00043)	0.98
TPHP	*y* = (0.09671 ± 0.00259)*x* + (0.00044 ± 0.00363)	0.98
T24DtBPP	*y* = (0.02457 ± 0.01208)*x* + (0.00427 ± 0.00523)	0.98

In addition to monitoring the inter-batch precision of the calibration range, intra- and inter-batch precision was assessed for three individual analytical standards spanning the calibration range (Table 5). Intra-batch precision was determined by calculating relative percent difference (RPD, %) for these analytical standards run in duplicate. Inter-batch precision was determined by relative standard deviation (RSD, %) of three analytical standards analyzed over *n* = 11 batches. Overall, the intra-batch precision was acceptable at the middle and high analytical standards but could be improved at the low level (0.010 ng/µL), which was at the LOQ for most analytes, excluding T24DtBPP. These trends were similarly observed for the inter-batch analyses as indicated by RSD (%), where the middle and high analytical standards had reasonably acceptable levels of precision for all analytes except T24DtBPP. This was an extremely troublesome analyte to work with, and precision could be remedied by finding an IS closer in structure and retention time to this analyte.

**Table 5:** Mean RPD, % values of duplicate analytical standards (intra-batch variation) and RSD, % values of multiple (*n* = 11) analytical standards analyzed over multiple days (inter-batch variation).

Analyte	*Intra-batch precision (n = 2 per batch)*			*Inter-batch precision (n = 11 batches)*		
	*Mean RPD, % over 11 batches*			*RSD, %*		
	0.010 ng/µL	0.25 ng/µL	1.0 ng/µL	0.010 ng/µL	0.25 ng/µL	1.0 ng/µL
TCPP	22	8.5	7.6	19	12	12
TDCPP	30.	12	21	51	32	37
TPHP	41	15	24	75	36	41
T24DtBPP	Not determined	21	13	Not determined	78	79

### LOD and LOQ

Limits of detection (LOD) and quantitation (LOQ) were calculated via two different methods. TCPP and T24DtBPP were present in the method blank. As such, the LOD values were calculated by determining the standard deviation of peak areas in method blanks (*n* = 10) and using Eq. (1) where *s* is the standard deviation, *t* is the t-value of *df* = 9*, and m* is the mean slope of the calibration curves (see Slope Sensitivity and Precision across the Calibration Range). The LOQ was the LOD × 3 (Table 6).

(1) LOD=(t×s)/m

**Table 6:** LOD and LOQ concentrations (ng/µL) for OPEs with analysis via GC/MS. LOQ is also reported as the absolute amount on-column.

Analyte	LOD (ng/µL)	LOQ (ng/µL)	LOQ (pg on-column)
TCPP	0.0040	0.010	10.
TDCPP	0.0050	0.010	10.
TPHP	0.0050	0.010	10.
T24DtBPP	0.11	0.32	320

TDCPP and TPHP were not detected in any method blank sample, so the LOD and LOQ for these compounds were calculated using the signal-to-noise (S/N) ratio. For both compounds, the LOQ was determined to be 0.010 ng/µL, where the S/N ≧ 8.

### Carryover

Carryover was assessed by analyzing a blank hexane/acetone (50/50, v/v) sample immediately after the analysis of the high calibration standard (1.0 ng/µL). Carryover was observed for three OPEs: TCPP, TPHP, and T24DtBPP when methanol was used as the rinsing solvent for the autosampler syringe. When this solvent was changed to acetone, carryover was no longer observed after the analysis of the 1.0 ng/µL standard. Carryover was observed in analytical batches only in which T24DtBPP was determined at very high levels above the calibrated region (> 3 ng/µL). When these samples were diluted, no carryover was observed.

### Interferences

Matrix effects were assessed for OPEs by extracting an HVAC air filter dust sample and spiking this sample with a known amount of OPEs. The % recovery of the matrix spike was determined and Eq. (2) used to calculate the percentage of matrix effects versus recoveries in the relatively clean chinchilla dust matrix. Because all matrix effects were much greater than 100% (Table 7), we concluded that ion enhancement was a significant issue. Thus, all air filter extracts were diluted 1:5 (see Sample preparation) to minimize matrix effects, and an internal standard was used to correct for the observed ion enhancement.

(2) Matrixeffect=[(Matrixspikerecovery,%)/(Chinchilladustspikerecovery,%)]×100

**Table 7:** Percent recoveries for matrix spikes at two different concentration levels and calculated matrix effect, %, when compared to a recovery in a clean matrix per Eq. (2).

Analyte	Mean Chinchilla Dust Spike Recovery, %	0.10 ng Spike Recovery, %	0.50 ng Spike Recovery, %	Mean Matrix Effect, %
TCPP	100.	326	196	261
TDCPP	120.	582	297	368
TPHP	114	347	223	249
T24DtBPP	128	211	122	130.

**Fig. 3:**
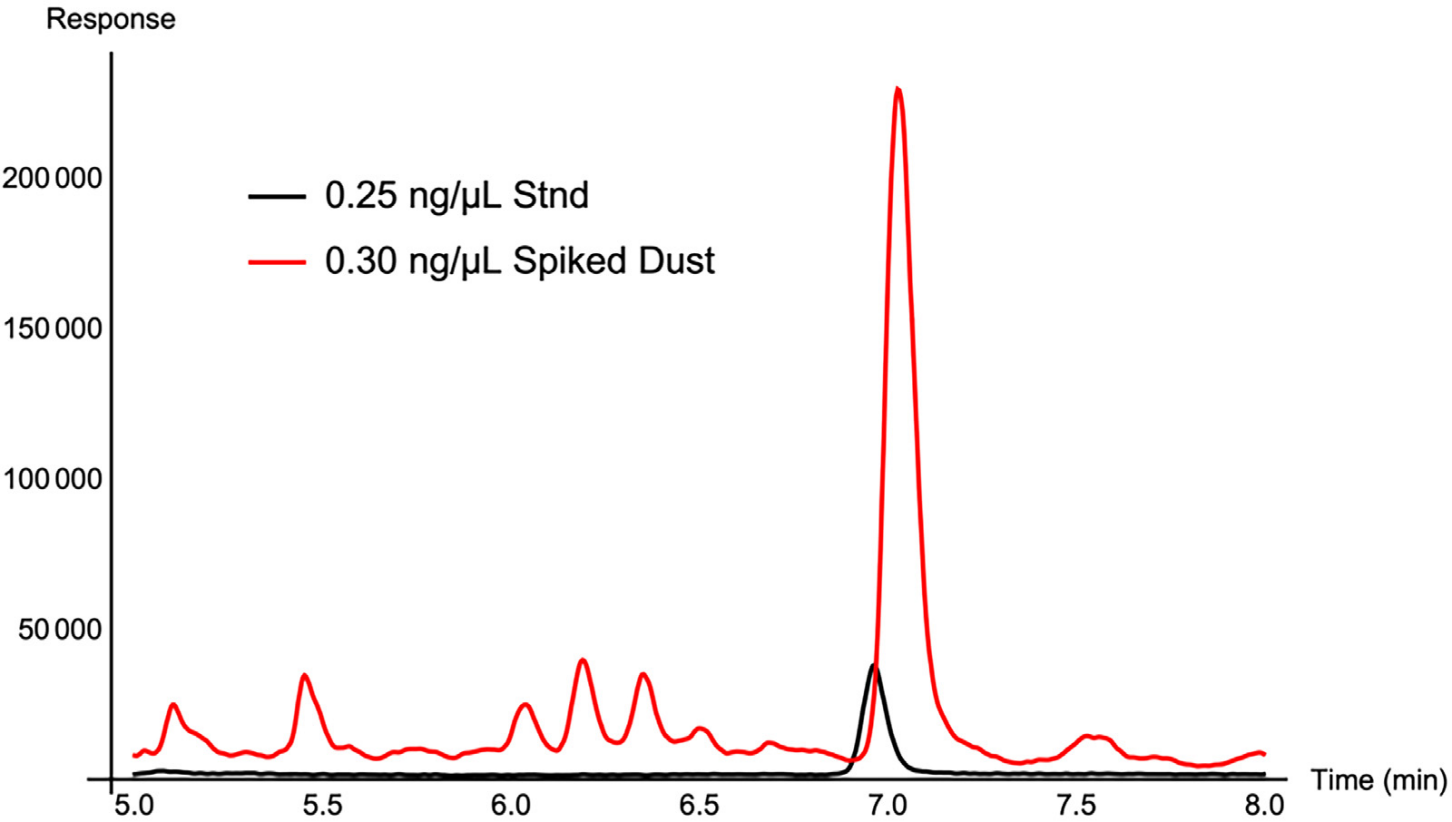
T24DtBPP peak within a 0.25 ng/µL analytical standard analyzed via LC/MS/MS (black trace) overlaid with a 0.30 ng/µL spiked chinchilla dust sample (red trace), demonstrating the significant and intense signal enhancement that was observed for the analysis of T24DtBPP when analyzed using LC/MS/MS.

Another important interference of the OPE method via GC/MS was the use of plastic consumables. Given that a majority of OPEs examined in this method are plasticizers, use of plastic laboratory consumables including microcentrifuge tubes caused a significant background signal (Fig. 3). Here, the T24DtBPP varied in an analytical standard prepared in glass versus a dust sample extracted in a plastic microcentrifuge tube and then analyzed via LC/MS/MS. Because of this large interference, only glass consumables were used and LC/MS/MS was not selected as the instrumental technique for quantitative analysis. LC/MS/MS likely had *more* interferences than GC/MS owing to the installation of plastic solvent lines within the instrument. To minimize any interference, all glassware used in the extraction of OPEs was silanized and baked in an oven at 250°C to avoid further contamination of samples.

To verify that air filters were an appropriate substrate for the sampling of dust within residential spaces, a background experiment was conducted to determine if air filters contained any background levels of OPEs. Here, chinchilla dust was added to a clean, unused HVAC air filter. This filter was stored in a plastic trash bag for one week to mimic the storage of collected air filter dust samples. The chinchilla dust was removed using the procedure described in Collection and removal of dust from HVAC air filters and extracted using the procedure outlined in Sample preparation. Results from this study demonstrated that there was not a significant concentration of OPEs in the chinchilla dust.

## Instrumentation and analysis of BFRs – LC/MS

The two BFRs quantitated in this study, TBBPA and TriBBPA, contain phenolic groups that require derivatization prior to GC/MS work. We have demonstrated previously in our laboratory that these compounds can be analyzed via GC/MS without derivatization but that their analytical sensitivity is greatly increased by derivatization via acetic anhydride. Using the LC/MS system allowed for this derivatization step to be avoided. Additionally, GC/MS analysis for the OPEs required dilution of the final extract owing to significant matrix effects (see LOD and LOQ) while the BFR sample extraction was undiluted owing to low concentrations of the two analytes found in the dust.

A Shimadzu LCMS-8030 system with an XBridge C18 column (50 × 2.1 mm, 3.5 µm) was used for all analyses. Mobile phase A was organic-free 18 MΩ DDI water with 0.1% (v/v) NH_4_OH and 2.5 mM ammonium formate. Mobile phase B was Optima LC/MS-grade methanol with 0.2% (v/v) NH_4_OH and 2.5 mM ammonium formate. The pH of the mobile phase was monitored daily as it was important that both solvents were ≧ 8 to ensure good peak shape. Mobile phase A was typically pH 9 while B was between 8 – 8.5. The injection volume was 5.0 µL, and the flow rate was 0.4000 mL/min. The column oven temperature was 40°C and gradient conditions were as follows: 5% B at 0.00 min (hold for 2.0 min), increase to 80% B (hold until 5.00 min), return to 5% B at 5.1 min (hold for 2.9 min) for a total time of 8.00 min.

**Fig. 4:**
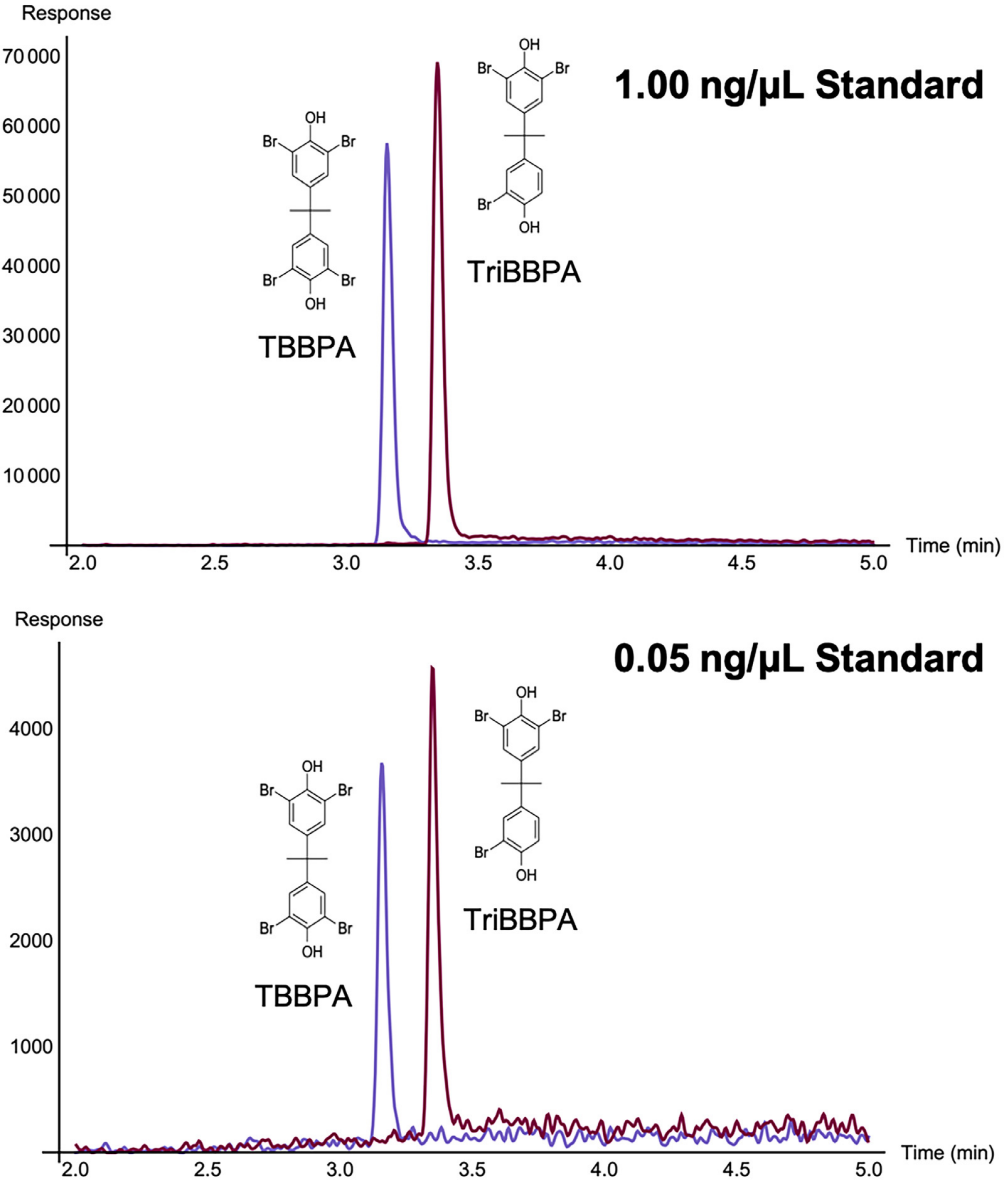
Extracted ion chromatograms (EIC) of (A) 1.0 ng/µL analytical standard of TBBPA (blue trace) and TriBBPA (maroon trace) and (B) 0.050 ng/µL analytical standard.

The ESI was operated in negative-ion mode with desolvation temperature set to 250°C, nebulizing gas of N_2_ flow was 1.50 L/min and drying gas (N_2_) flow of 15.00 L/min. TBBPA and TriBBPA were difficult compounds to fragment via CID for analysis via MRM, so the method utilized SIM for quantitation. Table 8 includes retention times (min), acquisition windows, quantitation ions (*m/z*), and confirmation ions (*m/z*). The dwell time was set to 38.0 ms. Extracted ion chromatograms (EICs) for 1.0 ng/µL and 0.050 ng/µL are shown in Fig. 4.

**Table 8:** Retention times (min), acquisition windows (min), and SIM ions (*m/z*) for TBBPA and TriBBPA.

Analyte	Retention Time (min)	Acquisition window (min)	Quantitation ion (*m/z*)	Confirmation ions (*m/z*)
TBBPA	3.25	2.00 – 5.00	542.80	544.80 540.70
TriBBPA	3.43	2.00 – 5.00	462.80	460.90 466.80

Slope sensitivity and precision across the calibration range

**Fig. 5:**
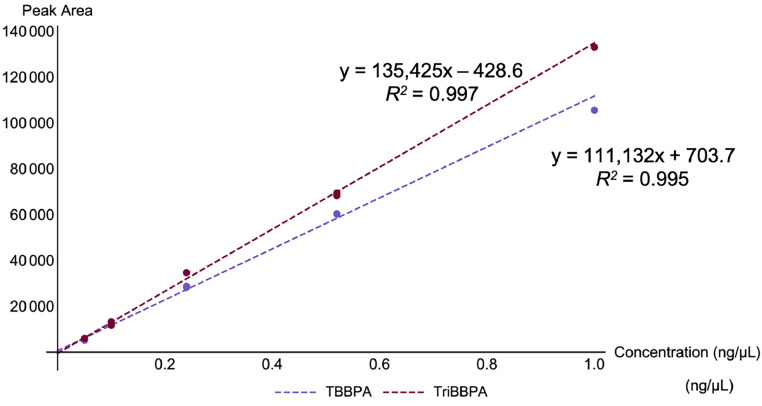
Examples of 1/*x* weighted linear regression equations for external calibration curves for the quantitation of TBBPA (blue) and TriBBPA (maroon).

Calibration standards for TBBPA and TriBBPA ranged from a low concentration level of 0.050 ng/µL (low level) to 1.0 ng/µL (high level). External, 1/*x* weighted calibration curves were utilized for quantitation (Fig. 5). Shimadzu LabSolutions software was used to generate all linear regression equations.

To monitor inter-day precision of the calibration curve, all equations used for quantitation of TBBPA and TriBBPA were recorded and 95% CIs calculated (Table 9). Regression lines were fairly consistent across multiple days. Mean *R^2^* values were 0.99 for both compounds. Additionally, intra- and inter-batch precision for the TBBPA and TriBBPA analytical standard 0.25 ng/µL was assessed. Intra-batch precision is reported as the RPD (%) for duplicate 0.25 ng/µL standards analyzed within the same batch whereas inter-batch precision is reported as the RSD (%) for replicate analyses of the 0.25 ng/µL standard over multiple (*n* = 4) batches.

**Table 9:** External calibration equations expressed as 95% CIs for *n* = 4 replicate analyses (df = 3) with intra- and inter-batch precision assessed for repeat analyses of the 0.25 ng/µL.

Analyte	Regression Equation	Mean *R^2^*	Mean Intra-Batch Precision (RPD, %)	Inter-batch Precision (RSD, %) for *n =* 4
TBBPA	*y* = (130840 ± 36314)*x* – (814 ± 915)	0.99	10.6	12.7
TriBBPA	*y* = (165697 ± 69323)*x* – (559 ± 840)	0.99	11.3	22.4

### LOD and LOQ

LOD and LOQ values were determined using S/N ratios (Table 10). However, the 0.050 ng/µL analytical standard was used as the lowest calibration standard.

**Table 10:** LOD and LOQ for TBBPA and TriBBPA analyzed via LC/MS. LOQ is reported in concentration units and absolute amount on-column.

Analyte	LOD (ng/µL)	LOQ (ng/µL)	LOQ (pg)
TBBPA	0.0050	0.010	50.
TriBBPA	0.0060	0.020	10. × 10^1^

### Carryover

Blank methanol samples were analyzed immediately after a high calibration standard (1.0 ng/µL) to determine if carryover was a concern for the method. All methanol blanks analyzed after these high calibration standards were devoid of TBBPA or TriBBPA signal, suggesting that carryover was not an issue for these analytes. Given that both compounds were detected at very low levels (all below 0.20 ng/µL), carryover at concentrations higher than 1.0 ng/µL was not assessed.

### Interferences

Matrix effects were assessed by spiking extracts of air filter dust with a known amount of TBBPA and TriBBPA and calculating the % recovery and comparing to % recovery of TBBPA and TriBBPA directly added to the air filter dust *prior* to extraction. Matrix effects were calculated using Eq. (2). The matrix effect (%) was determined to be 94% for TBBPA and 95% for TriBBPA. Given that these values were close to the expected 100%, it was concluded that matrix effects were not a significant concern for these analytes.

Though matrix effects were not significant in contributing to ion enhancement or suppression, it was critical to remove all suspended dust particles from the air filter extracts prior to LC/MS analysis. Any suspended dust in the final extract would cause clogged transfer lines or unacceptable chromatographic peak shapes. To ensure that all dust particles were removed, extracts were centrifuged at high speed with supernatants transferred to a clean PolySpring® autosampler vial insert. Additionally, sample extracts were never concentrated.

## Method validation

### Analytical batch and quality control

Air filter dust extracts for OPEs (GC/MS) and TBBPA and TriBBPA (LC/MS) from collected samples donated from area residents were analyzed with an analytical batch. All calibration standards were analyzed at the beginning of the batch with calibration check standards included in the middle and at the end of the batch to ensure accuracy and precision of the analytical standards.

On average, *n* = 5 air filter dust samples were analyzed in a batch with *n = 2* air filter extracts spiked and *n* = 2 air filter samples analyzed in duplicate to assess RPD (%). Spiked chinchilla dust samples (to assess % recovery without matrix effects) and a method blank (to assess carryover) were also included in every batch.

### Spike and recovery studies – OPEs

As mentioned above, the inclusion of spiked air filter and chinchilla dust samples were critical in assessing method accuracy. These matrices were spiked with known amounts of analyte during the method development and in every analyzed batch of household samples (see Analytical batch and quality control).

During the method development stage, chinchilla dust was used as a ‘clean’ environmental matrix. It was spiked at three levels (low, medium, and high) across the calibration range for each compound. Recovery results (with RSD, %) reported as 95% CIs from these spike and recovery studies are included in Table 11. Overall, these results demonstrated that the method was accurate across the calibration range when extracting from chinchilla dust.

**Table 11:** % Recoveries reported as 95% CIs where *n* = 5 replicates were included at the low and middle calibration levels and *n* = 7 replicates at the high calibration level for the 4 organophosphate esters of the study.

Analyte	Low – 0.080 ng on-column		Middle – 0.17 ng on-column		High – 0.57 ng on-column	
	Recovery, %	RSD, %	Recovery, %	RSD, %	Recovery, %	RSD, %
TCPP	97.7 ± 16.9	14.0	98.2 ± 4.5	3.7	103.0 ± 19.1	20.1
TDCPP	125.7 ± 19.5	12.5	114.9 ± 12.9	9.0	118.3 ± 24.7	22.6
TPHP	119.8 ± 19.8	13.3	110.8 ± 12.6	9.2	112.8 ± 23.9	22.9
T24DtBPP	82.6 ± 38.8	37.8	121.2 ± 30.9	20.6	165.9 ± 50.3	32.8

Air filter dust was also spiked in every analytical batch to demonstrate the accuracy of the method in the environmental matrix of interest. Air filter dust was spiked at 0.13 ng on-column (near the middle range, Table 11). At least one chinchilla dust sample was also spiked and extracted in every batch (see Analytical batch and quality control). Recovery results of air filter and chinchilla dust samples spiked in these analytical batches are shown in Table 12. Note that the recoveries for TCPP, TDCPP, and TPHP were within the desired range of 70 – 130% with narrow 95% CI and reasonable precision (RSD < 20%). These results demonstrated that the developed and applied method was accurate and precise for determination of TCPP, TDCPP, and TPHP in complex matrices like air filter dust. T24DtBPP had recoveries > 130% and RSD values < 50%. The results were consistent between the two matrices (air filter dust and chinchilla dust). We concede that a better IS other than HMB would be appropriate for this compound, which experiences significant matrix effects.

**Table 12:** Recovery results (as 95% CI) for chinchilla and HVAC air filter dust samples analyzed in each analytical batch (see Analytical batch and quality control). Each spike was equivalent to 0.13 ng on-column. For chinchilla dust spikes, these data were collected from *n* = 16 replicates. For FF dust, we had *n* = 25 replicates for TCPP, TDCPP, and TPHP and *n* = 20 replicates for T24DtBPP.

Analyte	Chinchilla Dust		HVAC Air Filter Dust	
	Recovery, %	RSD, %	Recovery, %	RSD, %
TCPP	108.0 ± 4.0	6.6	118.3 ± 9.3	18.9
TDCPP	112.5 ± 6.8	10.9	125.0 ± 7.2	13.8
TPHP	109.7 ± 7.2	11.8	118.8 ± 6.8	13.7
T24DtBPP	136.1 ± 35.3	46.6	136.0 ± 24.7	38.1

### Spike and recovery studies – TBBPA and TriBBPA

Similarly, the accuracy of the developed BFR method was assessed via spike and recovery methods for chinchilla and air filter dust. The chinchilla dust was spiked at three levels representing low (0.40 ng on-column), middle (0.85 ng on-column) and high (2.85 ng on-column) regions of the calibration range (Table 13). The % recoveries were within the desired range of 70 – 130% with RSD, % of < 20% indicating that the method was both accurate and precise for the chinchilla dust matrix.

**Table 13:** % Recoveries reported as 95% CIs where *n* = 5 replicates were included at the low and middle calibration levels and *n* = 7 replicates at the high calibration level for TBBPA and its derivative, TriBBPA.

Analyte	Low – 0.40 ng on-column		Middle – 0.85 ng on-column		High – 2.85 ng on-column	
	Recovery, %	RSD, %	Recovery, %	Analyte	Recovery, %	RSD, %
TBBPA	98.7 ± 14.6	11.9	117.0 ± 12.2	8.4	97.4 ± 14.1	15.7
TriBBPA	101.7 ± 13.5	8.3	104.4 ± 11.4	8.8	104.8 ± 9.3	9.6

After the initial validation of method development, spiked chinchilla dust samples were included with every batch along with spiked air filter dust samples. These chinchilla and air filter dust samples were spiked at the low point (0.43 ng on-column) of the calibration region. If these samples were concentrated four-fold to 1.70 ng on-column and injected onto the LC/MS system, the % recovery values were severely affected, indicating that matrix effects were significant for these compounds when samples were concentrated (Table 14). There were large 95% CIs for these results, however, only *n* = 2 samples were quantitated *without* the concentration step, so these results will likely improve with a larger sample size.

**Table 14:** Recovery results (as 95% CI) for chinchilla and HVAC air filter dust samples analyzed in each analytical batch (see Analytical batch and quality control). Each spike was equivalent to 0.43 ng on-column (*n* = 2 replicates) and then it was concentrated to assess matrix effects (1.70 ng on-column; *n* = 10 replicates).

Analyte	Chinchilla Dust				HVAC Air Filter Dust			
	% Recovery (0.43 ng)	RSD, %	% Recovery (1.70 ng – 4x conc)	RSD, %	% Recovery (0.43 ng)	RSD, %	% Recovery (1.70 ng – 4x conc)	RSD, %
TBBPA	75.5 ± 120.7	17.8	74.2 ± 22.6	42.5	96.0 ± 86.3	50.1	57.5 ± 13.4	32.5
TriBBPA	88.0 ± 101.6	12.9	46.7 ± 17.2	51.4	103.5 ± 41.9	22.5	27.8 ± 4.6	23.0

### Assessing precision via duplicate analyses

Intra-batch precision was assessed by analyzing two duplicate air filter dust samples in every analytical batch. Two 25-mg dust sub-samples from the same HVAC air filter were placed in separate vials and extracted in parallel. The concentration of all compounds (OPEs, TBBPA, and TriBBPA) were determined for both sub-samples, and the RPD (%) between the duplicate analyses were determined (Table 15). In general, an RPD < 20% indicated reasonable precision for the analyte. In these methods, the mean RPD values were < 20% for TCPP, TDCPP, TPHP, and TBBPA, demonstrating that extraction and analysis for duplicate samples is precise. The RPD (%) for T24DtBPP was slightly above 20%, but this precision was still deemed acceptable as this value was close to 20% and had a narrow 95% CI. It was not surprising that T24DtBPP had a slightly higher precision value (RPD, %) as we observed greater variability in quantitative levels of this analyte during our spike and recovery studies as well.

**Table 15:** Mean Relative Percent Difference (RPD), %, values with 95% CIs calculated for duplicate HVAC air filter dust samples analyzed within the same batch (intra-batch) and duplicate samples analyzed within different batches (inter-batch).

Analyte	Intra-Batch RPD, % (*n*)	Inter-Batch RPD, % (*n*)
TCPP	9.7 ± 4.0 (20)	34.4 ± 14.1 (5)
TDCPP	11.8 ± 6.9 (20)	30.7 ± 19.5 (5)
TPHP	14.5 ± 7.9 (20)	34.1 ± 21.4 (5)
T24DtBPP	21.0 ± 8.1 (20)	74.1 ± 46.3 (5)
TBBPA	16.4 ± 26.6 (3)	Not determined
TriBBPA	Not determined	Not determined

In addition to intra-batch precision, inter-batch precision was also examined and reported in Table 15. Here, duplicate samples were analyzed in different batches on different days and respective RPD values were determined. These samples were analyzed first in late November 2022 and again in late January 2023. As expected, RPD values were slightly higher when compared to intra-batch RPD values. The TCPP, TDCPP, and TPHP all had RPD < 40%. Inter-batch precision of T24DtBPP could be improved, but as we have previously expressed, this is a difficult compound to analyze.

We did not have any data to report for TriBBPA duplicates as this compound was not detected in any of our analyzed HVAC air filter dust samples. Given that we had such a small pool of duplicate data for TBBPA, replicate RPD (%) values were determined for TBBPA in addition to our intra-batch RPD. In replicate analyses, we injected the air filter dust extract on the LC/MS instrument twice three days apart. The replicate analyses here demonstrated an excellent level of precision with an RPD of 3.0%.

In summary, we present a validated method for the quantitation of selected OPEs and two brominated flame retardants via mass spectrometry following a facile extraction procedure from dust collected from HVAC air filters. We validated the method to assess accuracy, precision, and importantly, matrix effects. The method was applied for the analysis of 47 dust samples collected from 36 residential spaces in our companion report [1].

## Additional information

Household dust is an important sink for many hazardous components within the indoor environment including hazardous flame retardants (FRs) like polybrominated diphenyl ethers (PBDEs). PBDEs, a highly persistent class of chemicals, were once extensively used in consumer products including mattresses, carpet padding, personal electronics, and automotive cabin components [2]. Household dust is the primary route of exposure for PBDEs [3], contributing an average of 82% of a U.S. adult's exposure to this chemical class [4]. PBDEs have since been phased out owing to health concerns [5].

Other brominated flame retardants (BFRs) and organophosphate ester (OPE) flame retardants have been introduced as PBDE replacements. Many of these replacement compounds have also been identified in dust samples collected from home environments [6,7]. The levels in household dust for some of these replacement FRs have been evaluated extensively. Others have been evaluated in only a few studies; and there are concerns that these replacement compounds may too have negative health consequences, including neurotoxicity, reproductive toxicity, thyroid effects, and developmental toxicity [8,9].

## Ethics statements

The authors have read and followed the ethical requirements for publication in *MethodsX*. Neither human study participants, animals, nor social media platforms were involved in collecting the data presented here. No AI technology was utilized in the writing or preparation of the manuscript contents.

## CRediT authorship contribution statement

**Morgan L. Schachterle:** Methodology, Data curation, Validation, Writing – original draft, Writing – review & editing, Visualization. **Luis E. Lowe:** Methodology, Validation, Writing – review & editing. **Christopher R. Butler:** Methodology. **Allen M. Schoffstall:** Methodology. **Janel E. Owens:** Validation, Writing – original draft, Formal analysis, Visualization, Funding acquisition.

## Declaration of competing interest

The authors declare that they have no known competing financial interests or personal relationships that could have appeared to influence the work reported in this paper.

## Data Availability

Data will be made available on request.
